# Phospholipases A and Lysophospholipases in protozoan parasites

**DOI:** 10.15698/mic2023.10.805

**Published:** 2023-10-02

**Authors:** Perrine Hervé, Sarah Monic, Frédéric Bringaud, Loïc Rivière

**Affiliations:** 1 Université de Bordeaux, Microbiologie Fondamentale et Pathogénicité, CNRS UMR 5234, Bordeaux, France.

**Keywords:** phospholipases, lysophospholipases, protozoan, host-pathogen interactions, virulence factors, metabolism

## Abstract

Phospholipases (PLs) and Lysophospholipases (LysoPLs) are a diverse group of esterases responsible for phospholipid or lysophospholipid hydrolysis. They are involved in several biological processes, including lipid catabolism, modulation of the immune response and membrane maintenance. PLs are classified depending on their site of hydrolysis as PLA1, PLA2, PLC and PLD. In many pathogenic microorganisms, from bacteria to fungi, PLAs and LysoPLs have been described as critical virulence and/or pathogenicity factors. In protozoan parasites, a group containing major human and animal pathogens, growing literature show that PLAs and LysoPLs are also involved in the host infection. Their ubiquitous presence and role in host-pathogen interactions make them particularly interesting to study. In this review, we summarize the literature on PLAs and LysoPLs in several protozoan parasites of medical relevance, and discuss the growing interest for them as potential drug and vaccine targets.

## INTRODUCTION

**Figure 1:**
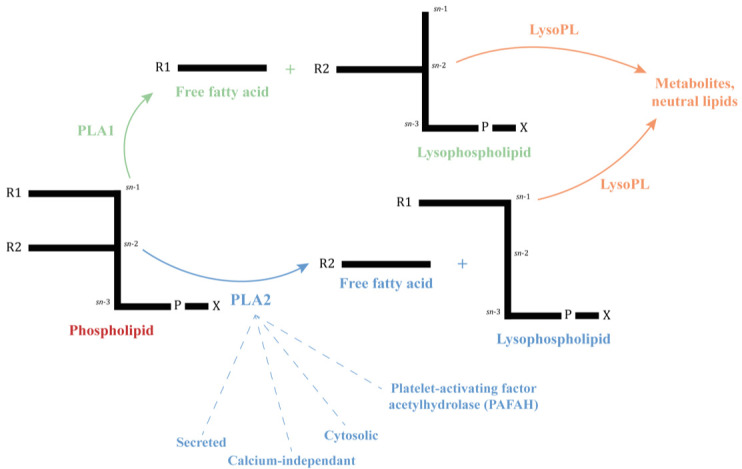
FIGURE 1: Simplified phospholipid structure and the site of cleavage of Phospholipases A and Lysophospholipases. A phospholipid consists of a glycerol-3-phosphate esterified with nonpolar fatty acids at its *sn*-1 (R1) and *sn*-2 (R2) positions and a polar headgroup (X = choline, ethanolamine, serine…) at its phosphoryl group. Phospholipases A are named A1 or A2 depending on their site on cleavage and the resulting molecules, indicated by arrows. Lysophospholipases act on lysophospholipids previously hydrolyzed by a PLA [1].

Phospholipases (PLs) and lysophospholipases (LysoPLs) are a widespread and complex group of lipolytic enzymes responsible for the hydrolysis of phospholipids and lysophospholipids, respectively. They are classified as A1, A2, C and D according to their site of cleavage (**Fig. 1**) [1]. These enzymes are involved in several biological processes including modulation of the immune response, cell signaling, membrane remodeling and lipid metabolism [2, 3]. Some of them, mainly phospholipases A (PLAs) and LysoPLs, play important roles in the virulence and pathogenicity of several groups of pathogens such as bacteria (*Pseudomonas, Ricktesia*) and fungi (*Candida*) [4, 5]. PLAs are also major toxins of venomous species [6]. For detailed information regarding PLs, please refer to Aloulou *et al.* [1].

Protozoans form a group of eukaryotic unicellular organisms that contain parasites responsible for severe and even fatal diseases worldwide. Their modes of transmission are diverse and include vectors (insects, gasteropods, annelids, etc.), contaminated food, water or soil, congenital transmission, blood transfusion or organ transplantation. Some of the diseases they cause are especially prevalent in developing countries due to lower standards of living and an advantageous climate for the presence of vectors. However, migration and climate change could favor the spread of these parasites globally. While some parasites such as the Apicomplexan *Plasmodium* spp. and *Toxoplasma* are heavily studied, others remain neglected despite the high risks they represent for susceptible populations [7, 8].

The importance of PLAs and LysoPLs for virulence in pathogenic species and their ability to manipulate the host immune response make them particularly interesting in the context of host – parasite interaction. Thanks to the sequencing of their genomes and the availability of many genomic resources, we know that most protozoan para-sites likely possess several *PLA* genes (https://veupathdb.org). However, recent studies are particularly focused on apicomplexan parasites, neglecting other major protozoan pathogens. Some characterized PLAs in pathogenic protozoan seem to be involved in the survival of the parasite inside its host and the establishment of an infection.

In this review, we focus on the different PLAs and LysoPLs characterized in several protozoan parasites of medical interest. We also discuss the relevance of these PLAs as potential drug and vaccine targets. The different parasites and enzymes described in this review are summarized in **Table 1** at the end of the conclusion.

**Table 1.** Characterized PLAs and LysoPLs of protozoan parasites.

**Protozoan parasite**	**Genome Database**	**Enzyme**	**Database Entry**	**Putative function s)**	**References**
*Toxoplasma gondii*	ToxoDB	TgLCAT	TGME49_272420	Facilitates egress from host cells	[10, 11]
		TgPL1	TGME49_232600	Supresses NO in macrophages, bradyzoite-to-tachyzoite reactivation	[12–14]
		TgPL2	TGME49_231370 aw	Apicoplast maintenance, regulation of fatty acids generation and PC/LPC levels	[15]
		TgPL3	TGME49_305140	Host cell invasion, rhoptry secretion, virulence in mice	[23]
*Plasmodium* sp.	PlasmoDB	PfLPL1	PF3D7_1476700	Conversion of heme to hemozoin	[34]
		PfLPL20	PF3D7_0702200	Hydrolysis of LPC	[108]
		PbPL	PBANKA_1128100	Sporozoites migration through the epithelial layers, merozoite release from hepatocytes	[36, 37]
		PfLCAT	PF3D7_0629300	Merozoite release from hepatocytes, egress in asexual blood stage	[38]
		PbPLA1	PBANKA_1423100	Merozoite release from hepatocytes	[39]
		PfPAPTL1/PN PLA1	PF3D7_0209100	Gametocyte induction and maturation, translocation of PfPLP2 to membrane periphery	[40, 41]
*Trypanosoma brucei*	TriTrypDB	TbPLA1	Tb927.1.4830	Synthesis of LysoPC metabolites	[53, 54]
		TbLysoPLA	Tb927.8.6390	Synthesis of LysoPC metabolites	[55, 56]
		TbGPI-PLA2	Tb927.7.6110	GPI precursor fatty acid remodeling	[61]
*Trypanosoma cruzi*		TcPLA1	TcCLB.510679.100	Plasma membrane remodeling	[72–74]
*Leishmania* sp.		LmPAFAH	LmjF35.3020	Regulation of PAF levels, survival in host macrophages, mediation of LysoPC levels	[80, 81]
		LbPLA1	LBRM2903_340037900	Hydrolysis of PC	[82]
		LdLip3	LdBPK_310860.1	Host fatty acids scavenging for lipid synthesis, tissue damage	[83]
*Cryptosporidium*	CryptoDB	No enzyme characterized			
*Entamoeba histolytica*	AmoebaDB				
*Naegleria fowleri*					
*Giardia*	GiardiaDB				
*Trichomonas vaginalis*	TrichDB				

## APICOMPLEXAN

Infections by Apicomplexan parasites represent huge health burdens worldwide. These parasites are characterized by the presence of the apicoplast, a plastid-like organelle which holds the lipid biosynthesis pathways [7].

### Toxoplasma gondii

*Toxoplasma gondii* is estimated to chronically infect one third of the world's population. This parasite can infect a wide range of warm-blooded animals, but its definitive hosts are felines. Although most infections are asymptomatic, congenital transmission or reactivation of latent disease in immunocompromised individuals such as AIDS, cancer or organ transplant patients can cause severe toxoplasmosis and represent significant public health burdens. Transmission usually occurs by ingestion of parasites or from an infected mother to the fetus. *Toxoplasma* parasites are present in three forms: an environment-resistant oocyst, a fast-replicating tachyzoite and a latent tissue cyst containing bradyzoites. *Toxoplasma* tachyzoites can replicate in a variety of nucleated cell types, while the chronic-stage bradyzoites persist as cysts within neurons or muscles [9].

Several PLAs of *Toxoplasma* have been characterized, including some important for the survival of the parasite in its host. TgLCAT (lecithin:cholesterol acyltransferase) produces cholesteryl esters through the transfer of phospholipid acyl groups (**Table 1**). The enzyme possesses both PLA2 and cholesteryl esterase activities and uses phosphatidylcholine (PC) as a substrate. TgLCAT is stored in dense granules and localizes to both the parasitophorous vacuole (PV) and plasma membrane following secretion from the dense granules. In a first study, parasites deficient for TgLCAT displayed a delay in replication and egress, suggesting a role in lipid membrane remodeling of both the parasite during replication and host cell during parasite escape. Mice infected with TgLCAT-null mutants showed reduced virulence, while parasites overexpressing TgLCAT were more virulent than the parental strain and resulted in earlier deaths [10]. In a later study using new strains, the authors confirmed its role in egress but showed that it has no contribution to replication *in vitro* or virulence *in vivo* [11].

Other PLA2s described include patatin-like phospholipases (PLPs), Ca^2+^-independent PLA2s. Three PLPs are known in *Toxoplasma* parasites. TgPL1 reduces the level of nitric oxide in activated macrophages to prevent its own degradation (**Table 1**) [12]. The enzyme lacks the catalytic serine and PLA2 activity, but the presence of other catalytic residues suggests a role in binding phospholipids or as a coenzyme for other PLA2 enzymes [13]. Mice infected with TgPL1-null mutants showed a greater resistance to toxoplasmic encephalitis and a change in cytokine levels during the chronic infection. The authors also observed a defect in bradyzoite-to-tachyzoite reactivation. The protein moves from within the parasite to the cyst wall and PV during tachyzoite-to-bradyzoite differentiation. Overall, TgPL1 seems to play a role in the maintenance of the infection in the host and toxoplasmosis encephalitis by altering cytokine production in the chronic phase [14].

A second PLP, TgPL2, is located at the apicoplast. This enzyme appeared essential for apicoplast maintenance and lipid homeostasis (**Table 1**). In parasites deficient for TgPL2 or with a mutated catalytic serine, the apicoplast was lost or became abnormal, leading to an impaired growth *in vitro* [15].

Ca^2+^-independent PLA2 activity associated with Ca^2+^- independent PLA1 activity was described in 2000, but it is unknown if one or several proteins were responsible for these activities [16].

Finally, PLA2 activity possibly linked to host cell invasion was first reported in 1989, when it was shown that incubation of the parasite with exogenous PLA2 from cobra (*Naja mossambica*) resulted in an increase of penetration in human fibroblasts. Meanwhile, PLA2 inhibition by 4-p-bromophenacyl bromide (4-BPB) decreased cell invasion, although the authors cautioned that 4-BPB seems to have a general toxic effect on parasites. Use of another PLA2 inhibitor, dihydroguaiaretic acid, also resulted in a decreased host cell invasion [17]. PLA2 activity could trigger arachidonic acid (AA) production from host cell phospholipids, which would alter the host cell membrane and facilitate parasite entry [18]. Incubation of extracellular *Toxoplasma* with exogenous PLA2 increased the presence of the rhoptry protein ROP1 in supernatant fractions, raising the possibility of a role of PLA2 in rhoptry protein secretion [19, 20]. Based on these results, another team confirmed the role of 4-BPB on THP1 monocyte cells. Furthermore, IFN-y treatment caused a reduction in host cell invasion, but whether that effect was direct or not on PLA is unclear [21, 22]. A PLP contributing to host cell invasion was recently reported. TgPL3 is located at the apical cap of the parasite. ΔTgPL3 parasites showed defects in invasion and rhoptry secretion; the authors hypothesize that TgPL3 has a role in either the mechanical release of rhoptry or in the signaling pathway leading to rhoptry release (**Table 1**). Mutation of the catalytic serine (S1409A parasites) also suppressed enzyme activity. Mice vaccination with ΔTgPL3 or S1409A parasites protected them against a subsequent infection with a lethal dose of *Toxoplasma* parasites [23].

### *Plasmodium* sp.

Malaria is the most common and deadliest human parasitic disease, and one of the most prevalent human infectious diseases. In 2021, 247 million cases and 619,000 deaths were reported [24]. 90% of deaths occur in Africa and mainly affect children under 5 and pregnant women. Parasites are transmitted by female mosquitoes of the *Anopheles* genus. Most cases and deaths are attributed to *P. falciparum*, but 4 other species of *Plasmodium* are also responsible for human infections: *P. vivax, P. ovale*, P. *malariae* and P. *knowlesi*. Parasites continuously evolve inside their hosts: the sporozoite insect form, once injected into a human, turns into a merozoites in hepatocytes. Merozoites are released into the bloodstream where they invade red blood cells (RBCs) and produce gametocytes. Mosquitoes ingest these gametocytes during a blood meal [25].

Cells infected by *Plasmodium* provide lipid precursors to the parasites, which are necessary for *de novo* synthesis of phospholipids and neutral lipids. These lipids are used for membrane maintenance and remodelling [26]. The presence of (lyso)phospholipases is therefore essential for maintenance of *Plasmodium* inside their hosts. More than 20 potential genes were identified in the genome of *Plasmodium* parasites [26, 27]. It was first shown that use of gentamicin and amikacin, aminoglycosides which bind to the 30S subunit of ribosomes, but are also known as PLA1 and PLA2 inhibitors, repressed *P. falciparum* growth in pretreated erythrocytes [28]. These enzymes could also be involved in the alteration of merozoite membrane phospholipid organization in the RBCs [29]. Furthermore, upon *P. falciparum* infection, both PLA2 and LysoPL activities were detected in human erythrocytes. LysoPL activity greatly increased in infected RBCs compared to non-infected ones and was much higher than PLA2 activity, suggesting that hydrolysis by LysoPLs is an important metabolic pathway for control of lysophospholipid levels. The authors never detected PLA2 activity from erythrocytes in uninfected conditions, but the origin of this activity (parasitic or infected RBCs) is unclear. PLA2 activity was inhibited *in vitro* by the antimalarial drugs chloroquine, quinine and artemether and LysoPL activity was inhibited by quinacrine and quinine, but the IC50 values for these inhibitions appear high and could be non-specific inhibitions due to high concentrations of the drugs. LysoPL activity was also inhibited by the sulfhydryl reagents p-hydroxymercuribenzoate and thimerosal, with IC50 values in much lower ranges [30, 31]. Later on, it was shown that P. falciparum parasites internalize host RBC peroxiredoxin 6 (PRDX6), an enzyme with PLA2 activity. The authors found that this host enzyme is used by the parasite for repair of lipid-peroxidation damages in P. falciparum blood stages and is essential for hemoglobin transport to the parasite food vacuole. Use of PLA2 inhibitors binding to PRDX6, such as methyl arachidonyl fluorophosphonate (MAFP), ATK and Daraplabid, blocked parasite growth at the trophozoite stage. Use of Daraplabid on WT mice arrested model rodent pathogen P. yoelii development, but had no effect in transgenic prd6x-/- mice, demonstrating a possible use of PRDX6 as a host drug target for Plasmodium infection control [32]. The PLA2 activity detected in infected RBCs could therefore be coming from the host and not Plasmodium [30, 32]. LysoPC from the host is also important for *P. falciparum* differentiation during infection. Restriction of the lipid, which occurs during malaria infection, led *in vitro* to metabolic adaptation of parasites, initiating sexual commitment and formation of gametocytes [33].

Several PLAs and LysoPLs have been identified in *Plasmodium* species. *Pf*LPL1 is a LysoPL localized in the endoplasmic reticulum (ER) at the early stages. After erythrocyte invasion, the enzyme moves to vesicular structures that are later incorporated into lipid bodies (**Table 1**). These bodies are found next to the food vacuole, where is stored hemozoin, the neutralized form of the toxic by-product heme*. Pf*LPL1 generates lipids necessary for hemozoin formation; downregulation of the enzyme resulted in disrupted lipid homeostasis and reduced levels of neutral lipids essential for conversion of toxic heme to hemozoin [34]. Another LysoPL from *P. falciparum, Pf*LPL20, hydrolyzes lysophosphatidylcholine (LysoPC) from the host to generate metabolites that can be used by the parasite for its growth. Like *Pf*LPL1, *Pf*LPL20 localizes to vesicular structures that later fuse in lipid bodies [35].

Two PLs were characterized in the rodent malaria model pathogen, *P. berghei*. First, PbPL is involved in sporozoite migration, localizes at their surface and possesses both PLA2 and LCAT activity (**Table 1**). Infection of mice with null-mutant sporozoites through mosquito bite resulted in a significant decrease in liver infectivity. Aa decrease in epithelial cell layers crossing was observed in PbPL knock-out parasites. Furthermore, an hemolytic assay showed that heterologously expressed PbPL was able to damage cell membranes, which the authors hypothesize could be caused by direct hydrolysis of membrane PC or through the production of LysoPC species, which possess membrane lysis activities [36]. In infected hepatocytes, PbPL is located at the PV membrane (PVM) where it plays a role in its disruption; PbPL-deficient parasites showed a defect in merozoite release from hepatocytes and oocysts [37]. This inefficient merozoite release was also reported in PbPL's ortholog in P. falciparum, PfLCAT. Lipidomics showed no significant changes in PC or LysoPC levels in PfLCAT-null mutants, but an accumulation of phosphatidylserine during egress was observed. PfLCAT-mutants also showed an inefficient egress in asexual blood stages (**Table 1**) [38]. A second enzyme from *P. berghei*, PbPLA1, is a phosphatidic acid preferring PLA1 (**Table 1**). The cytoplasmic enzyme is not expressed in sporozoites and is not essential in both mice infection and during mosquito stages. *Pla1*^*-*^ parasites, like PbPL-deficient parasites, showed a defect in merozoite release from hepatocytes [39].

Just as *Toxoplasma, Plasmodium* species possess PLPs. PfPATPL1 is expressed in the cytosol of asexual blood stage and gametocyte stages of *P. falciparum*. Depletion of the enzyme resulted in reduced efficiency of rounding up, egress of gametes after gametocyte activation as well as exflagellation of male gametes. Null-mutants for PfPATPL1 showed an impaired translocation of the perforin-like protein PfPLP2 to the membrane periphery. This translocation is necessary for destabilization of vacuolar and RBC membranes, allowing egress of gametes [40]. In a later study, another group described an effect of the PLP, which they re-named PNPLA1, in gametocyte induction rather than a deficiency in gametocytogenesis (**Table 1**) [41].

### Cryptosporidium

Cryptosporidiosis is a major diarrheal disease in young children and immunocompromised individuals worldwide. Food and water contaminated by feces or contact with infected animals and people are responsible for the transmission of parasites. *Cryptosprodium parvum* and *hominis* are the most prevalent species found during human infections. *Cryptosporidium* can complete its life cycle in a single host, and both asexual and sexual stages are found in the intestine of infected humans. The parasite does not possess an apicoplast or mitochondrial DNA and relies on its host metabolism to survive. After ingestion of oocysts, released sporozoites invade the small intestine and develop inside epithelial cells in an extracytoplasmic niche, where they maintain the infection [42].

Currently, not a single PLA/LysoPL gene is characterized in *Cryptosporidium*. However, secreted PLA2 (sPLA2) activity was observed in parasite lysate and could be involved in the invasion of the host enterocytes. Inhibition of PLA activity by 4-BPB or anti-sPLA2 antibodies resulted in a significant reduction in parasite reproduction in human enterocyte cell lines. Furthermore, treatment of enterocytes or *C. parvum* sporozoite with sPLA2 derived from cobra (*Naja naja*) venom before *in vitro* infection enhanced the number of intracellular parasites [43].

## KINETOPLASTIDS

Trypanosomatids include major pathogenic parasites responsible for neglected tropical diseases. These parasites are part of the Kinetoplastid group, organisms which possess within their single mitochondrion a concatenated and compacted network of circular DNA named kinetoplast [8].

### African Trypanosomes

Human and Animal African trypanosomiasis (HAT/AAT) are neglected tropical diseases caused by difference species of *Trypanosoma*. Two subspecies of *Trypanosoma brucei*, namely *T. b. gambiense* and *T. b. rhodesiense*, cause HAT or sleeping sickness. *T. b. brucei, T. vivax* and *T. congolense* are responsible for Nagana or AAT. The incidence of HAT has greatly decreased and WHO estimates an interruption of its transmission by 2030, but 70 million people remain at risk for the disease [44]. AAT cases are still very high, annually causing the death of 3 million livestock and losses of billions of US dollars in Africa. Parasites are transmitted by tsetse flies (*Glossina*) [44, 45]. African trypanosomes have a complex life cycle inside their hosts. The bloodstream mammalian form differentiates into procyclics when a tsetse fly takes a blood meal. In the insect, procyclics differentiate into epimastigotes and finally infective metacyclics, which are transmitted to mammals during a blood meal. In humans, infection occurs in two phases: an early hemolymphatic stage (fever, adenopathy…) and a late encephalitic stage in the CNS (neurologic troubles, biological clock disorders…) which eventually leads to death without adequate treatment. [44]. In animals, anemia and cachexia are the main symptoms, but abortion, decrease of milk production and weight loss are also observed [46].

Higher levels of PLAs activities were first observed in *T. brucei* and *T. congolense* compared to the nonpathogenic *T. lewisi* species present in rodents [47]. This activity was more important in bloodstream forms [48] and increased with parasite burden in tissue fluids and blood plasma of *T. brucei*-infected rabbits [49]. Bloodstream forms also generated phospholipids from exogenous lysophospholipids; this activity may help the parasite acquire fatty acids for the synthesis of the variant surface glycoprotein (VSG) membrane [50]. Indeed, a GPI-specific PLA may be involved in fatty acid remodelling leading to the biosynthesis of glycosylphosphatidylinositol (GPI), which anchors the trypanosome VSG to the plasma membrane [51]. Lastly, LysoPLA1 and PLA1 activities were eluted together from *T. brucei* soluble protein fractions and were indissociable, suggesting that the parasite possesses an enzyme displaying both activities [52].

Two *PLA1* genes have been characterized so far, both in *T. brucei*. TbPLA1 is a cytosolic enzyme displaying both PLA1 and LysoPLA1 activities and its preferred substrate is PC, resulting in the synthesis of LysoPC metabolites (**Table 1**) [53]. The enzyme levels and resulting metabolites were higher in the bloodstream forms compared to procyclics. Mutants for TbPLA1 were deficient in LysoPC synthesis. Homologues of this enzyme are found in the closely related *T. congolense* and *T. vivax*, but absent from more distant African trypanosomes or other Trypanosomatids. Its closest homologue is a putative PLA1 from *Soladis glossinidius*, a proteobacterium endosymbiont of the tsetse fly, suggesting that TbPLA1 may have been acquired through horizontal gene transfer [54].

TbLysoPLA is a recently characterized LysoPL. This secreted enzyme is localized in both the cytosol and glycosomes of bloodstream forms of *T. brucei.* TbLysoPLA showed PLA1 activity on non-natural phospholipids and PLA2 activity on natural phospholipids *in vitro* (**Table 1**). The enzyme acts on PC to produce LysoPC metabolites. TbLysoPLA is not essential for parasite survival but is probably highly immunogenic, since antibodies directed against it were generated during mice infection with *T. brucei gambiense* [55, 56].

PLA2 activity was also detected in African trypanosomes. In *T. brucei* bloodstream and procyclic forms, it may regulate Ca^2+^ entry in the parasite and catalyze AA release, which could be used to control eicosanoid acid production and cause changes in the host cell membrane [57, 58]. Furthermore, AA is the precursor for prostaglandin E2 (PGE2), a lipidic inflammatory mediator involved in mammalian immune responses [59]. However, this AA release from phospholipids can be PLA2-independent through a sequential sn-1 deacylation followed with hydrolysis by LysoPLA [60].

As previously mentioned, GPI-PLA activity was thought to be involved in trypanosome VSG remodeling [51]. A protein with GPI-PLA2 activity mediating the GPI precursor fatty acid remodeling was identified in procyclic T. brucei parasites. Deletion of the enzyme resulted in the suppression of GPI-PLA2 activity (Table 1). However, this enzyme was only characterized in procyclic parasites; VSG remodeling is an essential feature of bloodstream parasites, and whether or not it is essential in this form and its definitive role remain to be studied. The authors also caution that procyclic and bloodstream form parasites may use different genes for this same activity. Moreover, the authors hypothesize that another gene studied in this paper may encode for a GPI-PLA1 protein or a GPI-PLA2 activity in bloodstream parasites, but did not show it [61].

Anemia is a major symptom of *T. congolense* infection. A PLA2 from the parasite may be directly or indirectly responsible for the hemolytic activity observed during the course of infection, but the enzyme(s) involved is/are unidentified [62–66].

### Trypanosoma cruzi

*Trypanosoma cruzi* is responsible for Chagas disease or American trypanosomiasis. This anthropozoonosis is endemic in Latin America, although human migrations are leading to a global spread of the parasite. In endemic regions, the annual number of cases is estimated at 6 to 7 million, with many more at risk. *T. cruzi* is mainly transmitted by triatomine insects, also known as kissing bugs. The parasite has three main stages: a non-infective insect epimastigote, an infective trypomastigote and an intracellular replicating form inside mammals, the amastigote. The acute phase of infection is usually asymptomatic, with parasites invading and replicating in the peripheral circulation. Parasites can migrate to cardiac and digestive tissues where they contribute to the chronicity of the disease [67].

Phospholipases of *T. cruzi* are likely involved in global cellular lipid remodeling throughout the parasite's life cycle and temperature acclimation [68]. Both PLA1 and PLA2 membrane-associated activities on anionic phospholipids were observed in epimastigotes and may also be implicated in differentiation from trypomastigotes to amastigotes [69, 70]. Furthermore, the presence of exogenous PLA2 significantly increased association between *T. cruzi* and macrophages, but the origin of the enzyme was not specified. This association was reduced by use of PLA2 inhibitors such as 4-BPB, quinacrine and phentermine. A PLA2 could therefore be involved in macrophage invasion [71].

The previous observation in *T. brucei* that Ca^2+^ entry was regulated by generation of AA by PLA2 activity was also seen in *T. cruzi* amastigotes by addition of a PLA2 activator, melittin. In parallel, addition of the PLA2 inhibitor 3-(4-octadecyl)-benzoylacrylic acid (OBAA) decreased this influx [58].

TcPLA1 is the only PLA characterized in *T. cruzi*. It is responsible for PC degradation in autolysing parasites. The enzyme possesses both PLA1 and LysoPLA1 activities and its PLA1 activity is 20-fold higher in the infective trypomastigotes and amastigotes compared to non-infective epimastigotes [72]. In epimastigotes, TcPLA1 is found in the lysosome while in infective stages the activity of the enzyme is membrane-bound. Secretion of TcPLA1 was observed during differentiation from epimastigotes to trypomastigotes. *In vitro*, TcPLA1 generates DG, FFA and LPC, lipid secondary messengers that activate host cell protein kinase C which leads to an upregulation of parasite invasion [73]. Accumulation of bioactive products such as LysoPC could be controlled by the LysoPLA activity of the enzyme. Distinct levels of TcPLA1 activity were found between lethal and non-lethal strains of bloodstream trypomastigotes and use of antibodies directed against TcPLA1 reduced host cell invasion *in vitro* [74]

### *Leishmania* spp.

Leishmaniasis comprises several zoonotic disease endemic in many countries of Latin America and Asia. More than 20 species of *Leishmania* are responsible for the different clinical manifestations of leishmaniasis. The two main clinical manifestations are visceral leishmaniasis and cutaneous leishmaniasis. Hundreds of millions are at risk for each disease in almost 100 endemic countries. 700,000 to 1 million cases are reported every year. Parasites are transmitted by female phlebotomine sandflies. *Leishmania* exists in two forms: the mammalian stage amastigote and the insect stage promastigote. After infection, promastigotes parasites are phagocytosed by host macrophages, where they differentiate into infective amastigotes and multiply inside a parasitophorous vacuole. Parasites can persist for decades and reactivate in immunocompromised patients [75].

A first report showed that infection of macrophages by *L. amazonensis*, causative agent of cutaneous leishmaniasis, resulted in a significant increase of LysoPC in the host cell. LysoPC and AA are products of PLA2 activity on PC; production of PGE2 from AA is known to exacerbate *Leishmania* infection in macrophages [76, 77]. However, the origin of this activity (parasite or macrophage) was not specified [76]. PLA2 activity was detected in the supernatant and lysate of *L. amazonensis*. Treatment of amastigotes with PLA2 from *Crotalus durissus collilineatus* snake increased the growth of parasites within macrophages. Infection of BALB/c mice after promastigote treatment with PLA2 led to an increase in lesion size and larger regions of necrosis, as well as a higher density of inflammatory infiltrate. PLA2 activity inhibited IL-2 levels, a cytokine associated with a protective Th1 response and PGE2 generation. PLA2 activity in *Leishmania* parasites thus may be a mediator of cutaneous leishmaniasis [78]. In parallel, addition of the PLA2 inhibitors bromoenol lactone (BEL or MAFP) affected the survival of *L. (L.) amazonensis* promastigotes in culture. Treatment of intracellular *L. (L.) amazonensis* amastigotes with the same inhibitors resulted in decreased macrophage parasitism. Finally, BALB/c mice infected with *L. (L.) amazonensis* and treated with BEL presented a reduction of lesion size after 6 weeks of infection compared to non-treated infected mice. This decrease was associated with decreased skin parasitism [79].

Similarly, to *T. cruzi,* addition of melittin increased Ca^2+^ influx in in *L. donovani* promastigotes. Inhibition by OBAA reduced this influx [58].

*Leishamania* parasites possess in their genome a *LmHydrolPAFAH* gene (platelet-activating factor-acetylhydrolase) encoding a Ca^2+^-independent PLA2 which hydrolyzes platelet-activating factors (PAF), phosphoglycerides involved in inflammation (**Table 1**). The enzyme is found in the ER of promastigotes and amastigotes. Null-mutants for the enzyme showed no phenotype *in vitro* but a reduced virulence was observed in *L. major*-infected BALB/c mice. LmPAFAH may regulate levels of host platelet-activating factor (PAF), which could inhibit *Leishmania* survival in macrophages by NO activation. It may also be involved in mediation of levels of LysoPC to facilitate parasite survival [80]. The PAFAH protein from *L. major*, along with its ortholog in *T. cruzi* and *T. brucei*, is therefore an interesting drug and vaccine target [81].

LbPLA1 from *L. braziliensis* is responsible for PC hydrolysis in infective amastigotes and insect promastigotes. The purified recombinant protein exhibited PLA1 activity. The enzyme had a higher activity in amastigotes [82].

LdLip3 is a secreted lipase from *L. donovani*. The enzyme, found in both amastigote and promastigote forms, could act on host fatty acids in order to synthesize lipids for parasite growth and survival, as well as tissue damage associated with leishmaniasis (**Table 1**) [83].

## OTHER PROTOZOAN PARASITES OF MEDICAL RELEVANCE

Beside the more popular Apicomplexan and Trypanosomatids, some protozoan parasites such as amoebas or other flagellates cause severe disease worldwide.

### Entamoeba histolytica

Intestinal amebiasis caused by *Entamoeba histolytica* is a prevalent disease in developing countries with lower standards of sanitation. With 100 million cases each year, it is the fourth highest infectious parasite. 90% of infections remain asymptomatic. Two stages have been described: an active pathogenic trophozoite and a disseminating environmental amebic cyst. After ingestion of cysts through contaminated stools, released trophozoites invade the intestine and colonize the colon. In symptomatic cases, parasites interact with the epithelial cells and cause tissue damages [84].

It was first shown that antagonists of PLAs such as the PC analog Rosenthal's inhibitor and hydrocortisone were able to inhibit the cytolytic effects of trophozoite parasites *in vitro*, suggesting the involvement of PLAs in host cells lysis. [85]. The authors raised the hypothesis that *Entamoeba* PLA could directly act on the membrane of the target cell, or indirectly by the generation of cytotoxic lysocompounds. A more speculated argument could be the insertion of PLA in membranes, resulting in the activation of intracellular host PL and inducing autolysis in the presence of extracellular Ca^2+^ [85]. The same team also described Ca^2+^-dependent and Ca^2+^-independent PLAs. The first one was associated with membrane fractions while the second was mainly found in soluble fractions. A link was then established between these activities and cytolytic effects on host cells [86]. Hemolytic activity of *E. histolytica* was associated with a vesicular subcellular fraction called P30 and most likely due to a PLA [87–89]. In this fraction, both PLA1 and PLA2 as well as LysoPLA activities were identified [90]. Finally, it was observed that long-term axenic culture of *Entamoeba* caused a diminution of erythrophagocytosis, PLA2 and hemolytic activities. This was correlated with an impaired virulence of parasites [91]. The role of these PLAs in the virulence and pathogenicity of the parasite remains to be described. Although the genome of *E. histolytica* was sequenced in 2005 [92], no PLA-encoding gene has been characterized yet.

### Naegleria fowleri

Primary amoebic meningoencephalitis by the brain-eating amoeba *Naegleria fowleri* is a deadly, fulminating disease with a 98% mortality rate. The parasite possesses three forms: a pathogenic trophozoite, a transitory flagellated form in the presence of water and an environment-resistant cyst. After the parasite enters its host, most commonly through the nose when swimming, replicating trophozoites migrate to the olfactory bulb, where they enter the brain. Once in the CNS, the parasites proliferate and destroy tissue, ultimately causing death in 7 to 10 days [93].

In the early eighties it was shown that PLA, LysoPL and sphingomyelinase activities were substantially increased in the virulent *N. fowleri* cell line compared to non-pathogenic *Naegleria* spp. These activities were associated with cells and cell-free culture media. Moreover, the authors showed evidence that lipolytic activities were released in the media of the virulent cell line but not in culture media of non-pathogenic attenuated cells [94]. In a later study, the authors showed that PL-enriched culture of pathogenic *Naegleria* induced the degradation of human phospholipids [95]. This secreted activity could be at least partially responsible for the lipolytic activity observed in primary amoebic meningoencephalitis [96]. A Ca^2+^-independent PLA2 associated with membrane fraction could play a role in the parasite metabolism [97]. However, no *PLA* gene has been characterized yet.

### Giardia

With a prevalence up to 30% in developing countries and 300 million cases reported each year, giardiasis is one of the most common diarrheal disease worldwide. *G. duodenalis*, also known as *G. intestinalis* or *G. lamblia*, can infect all mammals. Infection occurs via the fecal-oral route, most commonly by ingestion of contaminated water. *Giardia* exists in two forms: a replicating trophozoite and a form of dissemination, the cyst. Once ingested cysts reach the stomach, the acidic pH and gastric enzymes induce an excystation. Parasites then migrate to the duodenum and differentiate into trophozoites. They adhere to intestinal epithelial cells where they multiply. Trophozoite can encyst back into quadrinucleated cysts, which are shed in the environment through the feces [98].

PLA activity has been detected in *in vitro* culture of *Giardia* [99, 100]. Like for *Entamoeba*, PLA2 activity was found in the P30 subcellular fraction. Cell fractionation suggested that two isoforms of PL sensitive to Rosenthal's inhibitor could be separated, one being membrane-bound and the other soluble [99]. The authors hypothesized that these activities could be responsible for the cytotoxicity of the parasite [99]. Interestingly, *Giardia intestinalis* is sensitive to the lipase inhibitor Tetrahydrolipstatin (Orlistat) *in vitro*, a drug which is also active on trypanosomes and *Plasmodium*. The target of Orlistat could be enzymes with a GXSXG motif, which is found in both PLA and fatty acid synthases [101].

### Trichomonas vaginalis

Trichomoniasis is the most common non-viral sexually transmitted infection in the world, with more than 250 million cases per year. *Trichomonas* only exists as a trophozoite. After infection, the parasite colonizes the human lower urogenital tract and adhere to host genital epithelial cells. Men are more commonly asymptomatic than women, but both are carriers and can manifest symptoms. [102]. Several studies suggest the role of a PLA2 in the pathogenicity of this parasite. PLA2 activity was measured in the vaginal fluids of pregnant women, which correlated with *Trichomonas* infection [103]. A material found in the soluble lytic fraction of *Trichomonas* had hemolytic activity and was able to degrade PC with lysed profiles similar to those obtained with a PLA2, suggesting that this lipase activity is PLA2-like [104]. PLA activity was detected in a subcellular fraction of *Trichomonas*. An hemolytic activity was correlated with the fraction, which decreased with addition of Rosenthal's inhibitor [105]. This observation is consistent with PLA1 and PLA2 activities from other parasites being involved in hemolysis [62–65, 87–89]. Indeed, PLA1 and PLA2 activities associated with soluble and membrane-bound fractions could induce indirect and direct hemolytic activities [105]. A PLA2 was isolated from a subcellular fraction, but solid functional genomics proofs need to be added. This eluted fraction caused hemolysis and was inhibited by Rosenthal's inhibitor [106]. The genome of *T. vaginalis* was sequenced in 2007 [107], but no *PLA* gene has been identified since then.

## CONCLUSION

Phospholipases and lysophospholipases play a crucial role in the host-parasite interactions by performing a variety of functions. For the last few years, there has been a clear interest in the study of these enzymes, with the discovery of new genes and new functions paving the way for further investigations. Studies have revealed their importance in the development and maintenance of protozoan pathogens inside their host, notably in common processes such as the invasion, host damages, production of active compounds and scavenging of metabolites for parasite survival. As of now, no single protozoan PLA/LysoPL has been shown to be essential. This could be explained by the fact that these parasites possess not only one but an arsenal of PLs that could compensate for the loss of one. Their roles in the virulence of these pathogens make them potential targets for drugs, vaccine or diagnostic development as was demonstrated for several protozoan or even host PLAs [23, 32, 55, 56, 80, 81]. The genome of protozoan parasites contains several different PL and LysoPL genes, but only a few have been characterized and studied yet, as summarized in **Table 1**, making their study a future necessity. Moreover, as we saw in this review there is, in some pathogens, a complete lack of knowledge at the gene level such as in intestinal parasites. It is crucial to continue deciphering the complexity of (lyso)phospholipases to improve our understanding of protozoan parasites and possibly eliminate them.

## Abbreviations:

PLs: – phospholipases,
LysoPLs: – lysophospholipases,
TgLCAT: – lecithin:cholesterol acyltransferase,
PC: – phosphatidylcholine,
PV: – parasitophorous vacuole,
PLPs: – patatin-like phospholipases,
4-BPB: – 4-p-bromophenacyl bromide,
AA: – arachidonic acid,
RBCs: – red blood cells,
PRDX6: – peroxiredoxin 6,
MAFP: – methyl arachidonyl fluorophosphonate,
LysoPC: – lysophosphatidylcholine,
sPLA2: – secreted PLA2,
HAT/AAT: – human and animal african trypanosomiasis,
VSG: – variant surface glycoprotein,
GPI: – glycosylphosphatidylinositol,
PGE2: – prostaglandin E2,
OBAA: – 3-(4-octadecyl)-benzoylacrylic acid,
PAF: – platelet-activating factor,
Orlistat: – tetrahydrolipstatin,
ER: – endoplasmic reticulum.
